# Sexual Compatibility Types in F_1_ Progenies of *Sclerospora graminicola*, the Causal Agent of Pearl Millet Downy Mildew

**DOI:** 10.3390/jof8060629

**Published:** 2022-06-13

**Authors:** Chandramani Raj, Rajan Sharma

**Affiliations:** 1 International Crops Research Institute for the Semi-Arid Tropics (ICRISAT), Patancheru, Hyderabad 502324, Telangana, India; chandramani.raj@icar.gov.in; 2 ICAR-Indian Institute of Sugarcane Research, Raebareili Road, Lucknow 226002, Uttar Pradesh, India

**Keywords:** mating types, homothallism, heterothallism, secondary homothallism, neuter

## Abstract

*Sclerospora graminicola* is primarily heterothallic in nature with two distinct mating types (G_1_ and G_2_); however, homothallism does exist in the pathogen populations. In this study, a cross was made between two self-sterile isolates (Sg 019, *Mat*-2, G_2_ × Sg 445-1, *Mat*-1, G_1_) of *S. graminicola* and a total of 39 F_1_ progenies were established. The study on sexual compatibility types in F_1_ progenies was conducted by crossing each F_1_ progeny with both the parents (Sg 445-1, *Mat*-1, G_1_; and Sg 019, *Mat*-2, G_2_). The results revealed the presence of four sexual compatibility types, *viz*. G_1_, G_2_, G_1_G_2_ and G_0_ (neuter) in the progenies. The G_1_G_2_ progenies that produced oospores with both the parents were found as self-fertile (homothallic) and self-sterile (heterothallic) types. Similarly, self-fertile parental type G_1_ and G_2_ progenies were designated as secondary homothallic, whereas self-sterile parental type G_1_ and G_2_ progenies were of heterothallic type. The result of the present study revealed Mendelian segregation of mating type locus in *S. graminicola* which indicates that sexual reproduction plays an important role in the evolution of new genetic recombinants in the pathogen. The study also helps in understanding the genetic structure of *S. graminicola* populations and potential for possible evolution of new virulences in the pathogen.

## 1. Introduction

Pearl millet [*Pennisetum glaucum* (L) R. Br.] is a choice crop of more than 90 million people cultivated on approximately 27 million hectares in the arid and semi-arid tropics of the world [1]. In India, mainly the states of Rajasthan, Gujarat, Haryana, Maharashtra, Uttar Pradesh, Karnataka and Andhra Pradesh produce 8.74 million tons of pearl millet. The crop is cultivated on 7.20 million hectares of land with a productivity of 1214 kg ha^−1^ [2]. Although average productivity of pearl millet in India has increased since the 1950s (305 kg ha^−1^) [3], it has also witnessed the devastating crop losses of up to 80% at periodic intervals caused by the downy mildew (DM) pathogen, *Sclerospora graminicola* [(Sacc). Schroet] [4]. The corresponding changes in the population structure of the pathogen over a period of time have played a key role in the destruction of the crop. The reason behind the evolution of new pathotype/s has been attributed to extreme selection pressure from the host along with sexual reproduction in *S. graminicola* populations [5]. 

The oospores formation in *S. graminicola* has been reported either through heterothallism, in which two self-sterile isolates having distinct sexual compatibility types, G_1_ and G_2_, fuse together [6,7], or through secondary homothallism in self-fertile isolates that contain the determinant of both compatibility types [8]. In general, one isolate produces functional antheridia and the other isolate forms oogonia during a reciprocal crossing between two self-sterile isolates and the evidence of relative sexuality within isolates determines the contribution of antheridia and oogonia by each parent [9]. However, the presence of multiple compatibility types has been reported in other oomycetes. Four compatibility types (A_1_, A_2_, A_1_A_2_ and neuter) have been observed in the F_1_ progenies of the crosses derived from two distinct mating type isolates (A_1_ × A_2_) of *Phytophthora* spp. [10,11]. The production of oospores in one mating type (G_2_) of *S. graminicola* isolate without fusion with any mating type [6] and no formation of oospores in isolate Sg 110-2 with any one of the designated mating types (G_1_ and G_2_) [12] indicated the presence of multiple compatibility types in *S. graminicola* [6]. Therefore, this study was planned to investigate the occurrence of self-sterile, self-fertile and neuter (sterile) isolates in *S. graminicola* to ascertain the multiple sexual compatibility types within the pathogen.

## 2. Materials and Methods

### 2.1. Collection and Maintenance of Isolates

A total of 52 isolates of *S. graminicola* were collected from different pearl millet growing areas of India during 1992 to 2012 (Table 1). The single zoospore isolates of each collection were established [12] and were maintained separately either on their original host or on another susceptible host in the isolation polyacrylic chambers (60 cm × 45 cm × 45 cm) in the glasshouse at ICRISAT, India. 

### 2.2. Identification of Self-Sterile or Self-Fertile Isolates

To identify the homothallic or heterothallic isolates, the single zoospore isolates-infected plants were allowed to mature for formation of oospores in separate isolation chambers. Necrotic leaf pieces from 2-month-old seedlings infected with each isolate were collected in brown paper bags, cut into 1-centimeter-long pieces, dried under shade and stored at room temperature (25 ± 2 °C) until further observation. The small leaf pieces were surface sterilized with NaOCl (2%) and washed thoroughly with sterilized distilled water. These leaf pieces were cleared by incubating them at 40 °C in NaOH (5%) for 12 to 16 h. Cleared leaf pieces were rinsed in distilled water and observed under a microscope using a 10× objective for the presence of oospores. Isolates which did not show oospore formation were selected as self-sterile isolates for further studies.

### 2.3. Selection of Highly Virulent Self-Sterile Isolate

The sporangial inocula of all the self-sterile heterothallic isolates were raised on seedlings of a highly susceptible genotype 7042 S in isolation chambers in the glasshouse. The sporangia from sporulating leaves were harvested in ice-cold distilled sterile water and spore concentration was adjusted to 1 × 10^6^ mL^−1^. Pot-grown seedlings of the pearl millet differential lines P 7-4, P 310-17, 700651, 7042 R, IP 18292, IP 18293 and 852 B and two known downy mildew (DM) susceptible lines—ICMP 451 and 7042 S—were spray-inoculated at coleoptile stage using an atomizer. The inoculated seedlings were incubated at 20 °C with >90% Relative Humidity (RH) for 20 h, and then transferred to greenhouse benches at 25 ± 2 °C and >90% RH for disease development for the next 2 weeks. DM incidence was recorded 14 days after inoculation as percentage of infected plants. The isolates with ≤10% disease incidence were considered avirulent and those with >50% disease incidence as virulent on the specific genotype.

### 2.4. Confirmations of Mating Type of Virulent Test Isolate (Sg 445-1)

The reference isolates Sg 018 (*Mat*-1, G_1_) and Sg 019 (*Mat*-2, G_2_) and test isolate Sg 445-1 (single zoospore selection from Sg 445) of *S. graminicola* were maintained separately on 7042 S. To detect the mating type of the test isolate, Sg 445-1 was crossed with both the reference mating type isolates (Sg 018 × Sg 445-1; and Sg 019 × Sg 445-1). Sporangial inoculum of each isolate (1 × 10^6^ sporangia mL^−1^) was prepared individually in ice-cold distilled sterile water. Sporangial suspensions of Sg 018 and Sg 445-1, and Sg 019 and Sg 445-1 were mixed in equal proportion (1:1) and spray inoculated on the highly susceptible pearl millet line 7042 S separately. The inoculated seedlings were incubated and transferred to isolation chambers. The infected seedlings were grown in the isolation chambers and allowed to mature. The necrotic tissues from these infected seedlings (>2 months old) were observed for oospore formation. 

### 2.5. Establishment of F_1_ Progenies from Oospores Generated from Sg 019 × Sg 445-1 Crosses

To generate progenies from F_1_ oospores (Sg 019 × Sg 445-1), infected leaf samples with oospores were dried in the shade, grinded and strained to make a fine powder. Oospores were checked again for their presence in the matured leaf powder. Sterilized potting mixture (soil, sand, and farmyard manure in a ratio of 3:2:2 by volume) was infested with oospore inoculum (20–25 g) and the pots (15 cm diameter) containing the infested mixture were sown with a susceptible genotype 7042 S (25 seeds per pot). Each pot was covered with a polythene bag and incubated at 40 °C for 3–4 days for rapid seed germination. Pots were transferred to isolation chambers in a glasshouse at 25 ± 2 °C to avoid any cross contamination from other isolates. Pots were watered adequately every day and observed regularly for DM symptoms on the seedlings. When the first infected seedling in a pot was noticed, it was removed from the pot and was transplanted into another pot containing sterilized soil and shifted to an isolation chamber. Sporangia from each seedling were maintained separately on 7042 S as an individual F_1_-progeny in isolation chambers at 25 ± 2 °C in the glasshouse. A total of 39 F_1_ progenies were established to determine sexual compatibility types in *S. graminicola*. Since infected seedlings occurred infrequently and rarely, each infected seedling was assumed to have infection from a single oospore.

### 2.6. Identification of Sexual Compatibility Types and Self-Sterile/Fertile Nature of F_1_ Progenies

To detect sexual compatibility types of F_1_ progenies, all the 39 F_1_ progenies derived from the cross Sg 019 × Sg 445-1 were crossed with both the parents (Sg 445-1, *Mat*-1, G1; and Sg 019, *Mat*-2, G2) separately. Sporangial inoculum (1 × 10^6^ sporangia mL^−1^) of each of the F_1_ progenies and both the parents was prepared separately in ice-cold distilled sterile water, mixed in equal proportion (1:1) and spray inoculated on the highly susceptible pearl millet line 7042 S separately. The inoculated seedlings were incubated, transferred to isolation chambers and the infected seedlings were allowed to mature for production of oospores. In addition, to identifying the self-sterile or self-fertile nature of F_1_ progenies, the single-zoospore infected plants were allowed to mature in separate isolation chambers and observed for the presence of oospores. 

## 3. Results

### 3.1. Selection of Self-Sterile Heterothallic Isolates

The 60-day-old, infected leaves of 52 single-zoosporic isolates of *S. graminicola* were checked for presence of oospores. No oospores were detected in 33 isolates, whereas oospores were formed by the remaining 19 isolates (Table 2). Isolates without oospores formation were designated as self-sterile or heterothallic while those producing oospores were designated as self-fertile or homothallic. Thus, a total of 33 heterothallic isolates were selected and the 19 homothallic isolates were excluded from the further studies.

### 3.2. Selection of Highly Virulent Self-Sterile Isolate

All the 33 self-sterile heterothallic isolates including reference mating type isolates Sg 018 (*Mat*-1/G_1_) and Sg 019 (*Mat*-2/G_2_) were screened on seven host differentials (P 7-4, P 310-17, 700651, 7042 R, IP 18292, IP 18293 and 852 B) and the two known DM susceptible lines (ICMP 451 and 7042 S). The screening identified Sg 445-1 as the most virulent isolate and Sg 018 and Sg 019, the two reference mating type isolates, as avirulent on specific genotypes; hence, they were selected for the crossing and generation of F_1_ progenies (Table 3). 

### 3.3. Confirmations of Mating Type of Virulent Test Isolate (Sg 445-1)

The cross between virulent test isolate Sg 445-1 with both the reference mating types Sg 018, *Mat*-1, G_1_ and Sg 019 *Mat*-2, G_2_ isolates (Sg 018 × Sg 445-1 and Sg 019 × Sg 445-1) yielded oospore production in the cross Sg 019 × Sg 445-1, whereas no oospore formations were recorded in Sg 018 × Sg 445-1. This indicated *Mat*-1/G_1_ mating type of Sg 445-1. Thus, two parents Sg 019 (avirulent) and Sg 445-1 (virulent) of different mating types were selected for crossing and generation of 39 F_1_ progenies.

### 3.4. Identification of Sexual Compatibility Types and Self-Sterile/Fertile Nature of F1 Progenies

A total of 39 F_1_ progenies were derived from the cross of Sg 019 *Mat*-2, G_2_ × Sg 445-1 *Mat*-1, G_1_. In contrast to the distinct mating types of the parents (G_1_ and G_2_), progenies were of four compatibility types *viz*. G_1_, G_2_, G_1_G_2_ and G_0_ (neuter) (Table 4). Of 39 F_1_ progenies, four belonged to G_1_, 13 to G_2_, 21 G_1_G_2_ and one to neuter categories (Table 4 and Table 5). Further, the self-fertile or self-sterile nature of all the 39 F_1_ progenies was evaluated on the basis of production of oospores. Among 21 G_1_G_2_ progenies, 19 supported self-production of oospores while 2 were free of any oospores in the matured leaves. Out of four G_1_ progenies, oospores were observed in three progenies and one was recorded as a non-oospore producer when selfed. Of the 13 G_2_ progenies, 7 supported self-production of oospores whereas no oospore formation was observed in the matured leaves infected with the remaining 6 F_1_ progenies. 

One unique neuter (G**_0_**) progeny was recorded as a non-oospore former, which was neither self-fertile nor produced oospore by crossing with any of the two parents. The F_1_ progenies which produced oospore by crossing with both the parents were designated as G_1_G_2._ Both self-sterile and self-fertile progenies were observed among G_1_G_2_s. In *S. graminicola*, it is reported that oospore formation is very low when isolates are selfed, whereas the number of oospores formed is quite high when the isolates of different mating types are crossed [6,12]. Similar observations were made in the present study. In the case of selfed G_1_G_2_ F_1_s, about 10 oospores were observed per leaf piece (1 cm^2^), whereas ~100-300 oospores were found when they were crossed with either of the parents. Thus, the 19 self-fertile (G_1_G_2_) progenies, which showed production of oospores with both parents, were designated as homothallic, while two self-sterile (G_1_G_2_) progenies were designated as heterothallic type (Table 5). Similarly, the self-fertile parental type G_1_ and G_2_ progenies were denoted as secondary homothallic whereas self-sterile parental type G**_1_** and G**_2_** progenies were of heterothallic type.

## 4. Discussion

The oospore formation in plant pathogenic oomycetes depends on the presence of two sexual compatibility types or their determinants [13,14,15,16,17]. In *S. graminicola*, two types of mating/compatibility types, *viz*. G_1_ and G_2_, have been proposed earlier [6,7,12] which are responsible for sexual reproduction between two self-sterile isolates, and within self-fertile isolates. Since sexual reproduction is dependent upon both compatibility types, it is speculated that the self-fertile isolates contain both compatibility types in the same seedling. The earlier studies [6,7,12] also reported self-fertile isolates and placed these isolates in G_2_ mating types tentatively and suggested that determination of sexual compatibility type in *S. graminicola* is likely to be complex and the nomenclature of G_1_/G_2_ compatibility types may not necessarily imply their distribution in a population. In addition, the neuter (sterile) type of *S. graminicola* isolate (Sg 110-2) was also observed [12], which failed to produce oospores with any of the parent isolates and was also placed under G_1_/G_2_ compatibility types. 

Since vegetative structures of oomycetes exist in diploidy level, the mating type alleles have been reported to be controlled by a single mating type locus in *Phytophthora* spp. [11,18,19] due to equal numbers of A_1_ or A_2_ types in the progenies. However, skewed numbers of one or the other mating types have also been reported [10,20,21,22]. Although normal Mendelian segregation of alleles expects four different combinations of alleles for a given locus in the progenies of heterozygous parents, inheritance of mating type alleles of a single locus has been explained in three different ways to explicate the almost equal ratios of A_1_ and A_2_ progenies in *Phytophthora* spp. [11,18,20]. 

In the first model, one mating type is represented by heterozygous (A/a) condition and the other in homozygous (a/a) condition at the mating type locus [20] which can yield only two types of sexual compatibility types in the offspring. However, inconsistent ratios in the progenies of heterozygous (A/a) and homozygous (a/a) parents have been reported in contrast to this model [15,22,23]. The second model suggests the presence of balanced lethal loci due to survival of only two genotypes A_1_ (M_1_/M_n_) and A_2_ (M_2_/M_n_) instead of the four different genotypes (M_1_/M_n_, M_2_/M_n_, M_1_/M_2_ or M_n_M_n_) in the progenies of A_1_ (M_1_/M_n_) and A_2_ (M_2_/M_n_) mating type parents in *Phytophthora infestans* [18]. The third model, a hybrid of the earlier two, explains the existence of ambiguous A_1_-A_2_ genotype in *P. parasitica*, which was consistent with the first model in which the A_1_ mating type was represented by heterozygous (M_A_/M_a_) and A_2_ in homozygous (M_a_/M_a_) conditions for the alleles at the mating-type locus [11]. In contrary to all three models, the present study revealed four different compatibility types (4G_1_, 13G_2_, 21G_1_G_2_ and one G_0_, neuter) in 39 F_1_ progenies from the cross of two distinct self-sterile heterothallic parents (Sg 445-1 *Mat*-1, G_1_ × Sg 019 *Mat*-2, G_2_) that indicated normal Mendelian segregation of mating types (Table 6) in *S. graminicola*. In the earlier studies [6,12], four different compatibility types were also noticed in *S. graminicola* though all the progenies were accommodated in G_1_/G_2_ compatibility types either due to skewed distribution of mating types or lack of nomenclature in *S. graminicola*. The discussed three models were found inadequate to explain the usual segregation in *S. graminicola* and unequal ratio of G_1_:G_2_ along with ambiguous G_1_G_2_ sexual compatibility types. Therefore, an alternative scheme for mating-type determination was considered and the segregation could be speculated due to presence of mating type alleles in heterozygous state in both parents [G_1_g_1_ (*Mat*-1) for G_1_ and G_2_g_2_ (*Mat*-2) for G_2_] at the same locus. In *Phytophthora*, isolates forming oospores only with the A_1_ or A_2_ testers are designated as A_2_ and A_1_, respectively, whereas the isolates which can form oospores with both A_1_ and A_2_ testers are designated as A_1_A_2_ and those that fail to form oospores are designated as A_0_ (sterile or neuter) [24] which supports the results of this study.

The mating system plays an important role in the evolution of plant pathogens during strong selection pressure from the resistant host or chemical control measures or harsh environmental conditions [25,26]. In oomycetes, the predominant co-existence of two mating types (G_1_ and G_2_ or A_1_ and A_2_) [6,7,11,12,18,19] and generation of multiple compatibility types (A_1_, A_2_, A_1_A_2_ and neuter) in the F_1_ progenies upon sexual reproduction between two distinct mating types (A_1_ × A_2_) [10,11] might provide advantage to pathogens during unfavorable conditions. *Sclerospora graminicola* has a high outcrossing capacity which renders the pathogen to evolve into new pathotype/s upon selection pressure and helps in adaptation to different ecosystems [12]. Therefore, effective management of downy mildew pathogen in pearl millet would be targeted towards understanding the change in population structure, particularly virulence pattern, and its utilization in resistance-breeding programs for the development of resistant cultivars.

## Figures and Tables

**Table 1:** Sources of *Sclerospora graminicola* isolates collected from different pearl millet growing states of India.

Identity	Location	State	Year	Maintenance Host
Sg 018	Patancheru	Telangana	1992	7042 S
Sg 019	Patancheru	Telangana	1992	7042 S
Sg 021	Ahmednagar	Maharashtra	1993	7042 S
Sg 048	Mysore	Karnataka	1994	852 B
Sg 139	Jodhpur	Rajasthan	1997	Nokha Local
Sg 150	Jalna	Maharashtra	1997	834 B
Sg 151	Durgapura	Rajasthan	1997	Nokha Local
Sg 153	Patancheru	Telangana	1997	843 B
Sg 200	Jamnagar	Gujarat	1998	ICMP 451
Sg 212	Durgapura	Rajasthan	1998	ICMP 451
Sg 298	IARI	New Delhi	1999	W 504-1-1
Sg 334	Bhiwani	Haryana	2001	7042 S
Sg 384	Barmer	Rajasthan	2003	ICMP 451
Sg 409	Patancheru	Telangana	2004	PMB 11571-2
Sg 431	Patancheru	Telangana	2005	7042 S
Sg 445	Banaskantha	Gujarat	2005	Pioneer 7777
Sg 457	Sujnapur, Jaipur	Rajasthan	2006	ICMP 451
Sg 492	Iglas	Uttar Pradesh	2007	ICMP 451
Sg 510	Badaun	Uttar Pradesh	2008	7042 S
Sg 519	Rewari	Haryana	2009	7042 S
Sg 520	Bhiwani	Haryana	2009	7042 S
Sg 521	Rewari	Haryana	2009	7042 S
Sg 526	Jodhpur	Rajasthan	2009	7042 S
Sg 528	CAZRI, Jodhpur	Rajasthan	2009	7042 S
Sg 529	CAZRI, Jodhpur	Rajasthan	2009	7042 S
Sg 530	Karodi, Aurangabad	Maharashtra	2009	7042 S
Sg 531	Nashik	Maharashtra	2009	7042 S
Sg 532	Srirampur, Ahmednagar	Maharashtra	2009	7042 S
Sg 533	Newasa, Ahmednagar	Maharashtra	2009	7042 S
Sg 535	Gangapur, Aurangabad	Maharashtra	2009	7042 S
Sg 540	Jambal, Aurangabad	Maharashtra	2010	7042 S
Sg 541	Pimpalgaon, Aurangabad	Maharashtra	2010	7042 S
Sg 542	Aurangabad	Maharashtra	2010	7042 S
Sg 543	Aurangabad	Maharashtra	2010	7042 S
Sg 544	Aurangabad	Maharashtra	2010	7042 S
Sg 545	Aurangabad	Maharashtra	2010	7042 S
Sg 546	Tanda, Aurangabad	Maharashtra	2010	7042 S
Sg 547	Jalna	Maharashtra	2010	7042 S
Sg 548	Dakkalgaon, Jalna	Maharashtra	2010	7042 S
Sg 549	Hathnur, Aurangabad	Maharashtra	2010	7042 S
Sg 550	Kannad, Aurangabad	Maharashtra	2010	7042 S
Sg 551	Chalisgaon, Jalgaon	Maharashtra	2010	7042 S
Sg 552	Sindhkheda, Dhule	Maharashtra	2010	7042 S
Sg 553	Dondaicha, Dhule	Maharashtra	2010	7042 S
Sg 554	Indave, Dhule	Maharashtra	2010	7042 S
Sg 555	NARP, Aurangabad	Maharashtra	2010	7042 S
Sg 556	Kothigaon, Banaskantha	Gujarat	2010	7042 S
Sg 557	Lodhnoor, Banaskantha	Gujarat	2010	7042 S
Sg 558	Gagana, Banaskantha	Gujarat	2010	7042 S
Sg 559	Jamdi, Banaskantha	Gujarat	2010	7042 S
Sg 560	SK Nagar, Banaskantha	Gujarat	2010	7042 S
Sg 561	IARI	New Delhi	2010	ICMP 451

**Table 2:** Observation on oospore formation in 52 selfed *Sclerospora graminicola* isolates.

S.No.	Isolate No.	Oospore Formation		S.N.	Isolate No.	Oospore Formation	
		No Oospore	Oospores			No Oospore	Oospores
1	Sg 018	√		28	Sg 532	√	
2	Sg.019	√		29	Sg 533	√	
3	Sg 021		√	30	Sg.535	√	
4	Sg 048	√		31	Sg 540	√	
5	Sg 139	√		32	Sg 541		√
6	Sg 150	√		33	Sg 542	√	
7	Sg 151	√		34	Sg 543	√	
8	Sg 153		√	35	Sg 544	√	
9	Sg 200	√		36	Sg 545		√
10	Sg 212	√		37	Sg 546		√
11	Sg 298	√		38	Sg 547	√	
12	Sg 334		√	39	Sg 548		√
13	Sg 384	√		40	Sg 549	√	
14	Sg 409		√	41	Sg 550		√
15	Sg 431	√		42	Sg 551		√
16	Sg 445	√		43	Sg 552	√	
17	Sg 457	√		44	Sg 553	√	
18	Sg 492	√		45	Sg 554	√	
19	Sg 510		√	46	Sg 555	√	
20	Sg 519		√	47	Sg 556	√	
21	Sg.520		√	48	Sg 557	√	
22	Sg 521		√	49	Sg 558		√
23	Sg 526	√		50	Sg 559		√
24	Sg 528	√		51	Sg 560		√
25	Sg 529	√		52	Sg 561		√
26	Sg 530	√					
27	Sg 531		√				

**Table 3:** Differential reaction of the isolates selected for developing F_1_ progenies.

Pathotype	Mating Type	Percent Disease Incidence on Host Differential Lines								
		700651	7042 R	7042 S	852 B	ICMP451	IP18292	IP18293	P310-17	P7-4
Sg 018	*Mat-1*	4	47	97	0	94	0	4	0	8
Sg 019	*Mat-2*	0	38	95	0	91	0	0	0	3
Sg 445	?	53	75	100	100	100	80	46	63	86

**Table 4:** Determination of sexual compatibility types of F**_1_** progenies based on oospores formation with Sg 445, *Mat*-1 (G_1_) and Sg 019, *Mat*-2 (G_2_).

Population	Oospore Formation with		Mating Type of Population	Self-Fertile/ Sterile	Remarks
	Sg 445-1 (G_1_)	Sg 019 (G_2_)			
P_1_	N	Y	G_1_	N	Heterothallic
P_5_	Y	N	G_2_	N	Heterothallic
P_6_	Y	Y	G_1_G_2_	Y	Homothallic
P_7_	Y	N	G_2_	N	Heterothallic
P_8_	Y	N	G_2_	N	Heterothallic
P_10_	N	Y	G_1_	Y	Secondary homothallic
P_11_	Y	Y	G_1_G_2_	Y	Homothallic
P_12_	Y	N	G_2_	Y	Secondary homothallic
P_14_	Y	Y	G_1_G_2_	Y	Homothallic
P_18_	Y	Y	G_1_G_2_	Y	Homothallic
P_19_	Y	N	G_2_	N	Heterothallic
P_20_	N	Y	G_1_	Y	Secondary homothallic
P_21_	Y	N	G_2_	N	Heterothallic
P_22_	Y	N	G_2_	Y	Secondary homothallic
P_23_	Y	N	G_2_	N	Heterothallic
P_24_	Y	Y	G_1_G_2_	Y	Homothallic
P_25_	Y	Y	G_1_G_2_	N	Heterothallic
P_26_	Y	Y	G_1_G_2_	Y	Homothallic
P_27_	Y	Y	G_1_G_2_	Y	Homothallic
P_28_	Y	N	G_2_	Y	Secondary homothallic
P_29_	Y	Y	G_1_G_2_	N	Heterothallic
P_30_	N	N	Neutral	N	Neuter
P_31_	Y	Y	G_1_G_2_	Y	Homothallic
P_32_	Y	N	G_2_	Y	Secondary homothallic
P_33_	Y	N	G_2_	Y	Secondary homothallic
P_34_	Y	Y	G_1_G_2_	Y	Homothallic
P_35_	Y	Y	G_1_G_2_	Y	Homothallic
P_36_	Y	Y	G_1_G_2_	Y	Homothallic
P_37_	Y	N	G_2_	Y	Secondary homothallic
P_38_	Y	Y	G_1_G_2_	Y	Homothallic
P_39_	N	Y	G_1_	Y	Secondary homothallic
P_40_	Y	Y	G_1_G_2_	Y	Homothallic
P_41_	Y	N	G_2_	Y	Secondary homothallic
P_42_	Y	Y	G_1_G_2_	Y	Homothallic
P_43_	Y	Y	G_1_G_2_	Y	Homothallic
P_44_	Y	Y	G_1_G_2_	Y	Homothallic
P_45_	Y	Y	G_1_G_2_	Y	Homothallic
P_46_	Y	Y	G_1_G_2_	Y	Homothallic
P_47_	Y	Y	G_1_G_2_	Y	Homothallic

N = no oospore, Y = oospores formed.

**Table 5:** Summary of determination of sexual compatibility types of F**_1_** populations based on oospore formation with Sg 445, *Mat*-1(G_1_) and Sg 019, *Mat*-2 (G_2_).

No. of Progenies	Self-Fertile	Oospore Formation		Compatibility Types	Remarks
		Sg 445-1	Sg 019		
19	Y	Y	Y	G_1_ G_2_	Homothallic
2	N	Y	Y	G_1_ G_2_	Heterothallic
3	Y	N	Y	G_1_	Secondary homothallic
1	N	N	Y	G_1_	Heterothallic
6	N	Y	N	G_2_	Heterothallic
7	Y	Y	N	G_2_	Secondary homothallic
1	N	N	N	G_0_	Neuter

N = no oospore, Y = oospores formed.

**Table 6:** Mendelian segregation of sexual compatibility types in two distinct self-sterile heterothallic parents (Sg 445-1, *Mat*-1, G_1_ × Sg 019, *Mat*-2, G_2_) of *Sclerospora graminicola*.

G_1_g_1_ (*Mat*-1) × G_2_g_2_ (*Mat*-2)			
	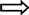	G_2_	g_2_
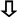			
G_1_		G_1_ G_2_ (*Mat*-1*/Mat*-2)	G_1_ g_2_ (*Mat*-1)
g_1_		G_2_ g_1_ (*Mat*-2)	g_1_ g_2_ (G_0_, Neuter)

## Data Availability

Data available at http://dataverse.icrisat.org/privateurl.xhtml?token=f0c65f21-6a85-4b3f-901d-1f03703d6311, accessed on 18 March 2022.
